# On the Komlós–Révész SLLN for Ψ-Mixing Sequences

**DOI:** 10.3390/e27010036

**Published:** 2025-01-04

**Authors:** Zbigniew S. Szewczak

**Affiliations:** Faculty of Mathematics and Computer Science, Nicolaus Copernicus University, ul. Chopina 12/18, 87-100 Toruń, Poland; zssz@mat.umk.pl

**Keywords:** strong laws, Komlós–Révész law, *ψ*-mixing, dependent Cramér model, 60F15, 11N05

## Abstract

The Komlós–Révész strong law of large numbers (SLLN) is proved for ψ-mixing sequences without a rate assumption.

## 1. Introduction and Result

Let ${\left\{X_{k}\right\}}_{k\in N},$ N={1,2,3,…}, be a sequence of random variables defined on a probability space (Ω,F,P) and ${∥X∥}_{p}^{p}={E(|X|}^{p})$. It was proved by Komlós and Révész in [1] that for centered, independent random variables $X_{k}$,

(1) $$\frac{\sum_{k=1}^{n}{∥X_{k}∥}_{2}^{-2}X_{k}}{\sum_{k=1}^{n}{∥X_{k}∥}_{2}^{-2}}\to_{n}0,\;a.s.$$

provided that $\sum_{k=1}^{\infty }{∥X_{k}∥}_{2}^{-2}$ diverges (see also [2], p. 75 and Ex. 13 on p. 137 in [3]). If $E(X_{k})=m,$ k≥1, then we have strong consistency:

$$\frac{\sum_{k=1}^{n}{∥X_{k}-m∥}_{2}^{-2}X_{k}}{\sum_{k=1}^{n}{∥X_{k}-m∥}_{2}^{-2}}\to_{n}m,\;a.s.$$

and it was proved that this estimator has minimal variance. Nevertheless, as observed in the preface of [4], *“For many phenomena in the real world, the observations are not independent…”*, so the question on (1) is intriguing when there is a lack of independence. In [5], the Komlós–Révész theorem is investigated for pairwise independence by the method of subsequences. For martingale difference sequences in $L^{p}$, p∈(1,2], by the Doob theorem (see Th. 2 on p. 246 in [3]), it is obtained in [6]. In [7], the case p∈(0,1) and p>2 is also discussed for negatively dependent and mixing sequences. In the latter, some rate on mixing is assumed, so this paper aims to remove this restriction.

Let σ-fields A,B satisfy A,B⊂F. Recall the following strong measure of dependence:

$$\psi \left(A,B\right)=\sup|\frac{P(B\cap A)}{P(B)\cdot P(A)}-1|;\;P\left(B\right)\cdot P\left(A\right)>0,\;A\in A,B\in B.$$

It is well known that (see p. 124 in Vol. I, [4])

$$\psi_{m}=\underset{J\geq 1}{\sup}\psi \left(F_{\;\;1}^{\;J},F_{J+m}^{\infty }\right)=\underset{J\geq 1}{\sup}\sup\frac{\|E\left(g|F_{\;\;1}^{\;J}\right)-E\left(g\right){\|}_{\infty }}{{\left\|g\right\|}_{1}},$$

where $F_{\;k}^{\;m}$ denotes σ–field generated by $X_{k},X_{k+1},\ldots ,X_{m}$, m∈Z and the inner sup is taken over $g\in L_{\mathrm{real}}^{1}\left(F_{J+m}^{\infty }\right)$. We say that $\left\{X_{k}\right\}$ is ψ-mixing if $\lim_{m\to \infty }\psi_{m}=0$. It is worth noticing that Kesten and O’Brien and Bradley gave examples of ψ–mixing sequences with an arbitrary rate of mixing (see Ch. 3 and Ch. 26 in [4]).

The following is the main result in this paper.

**Theorem 1.**  *Suppose $\left\{X_{k}\right\}$ is ψ–mixing. Let $\left\{a_{k}\right\}$ be a sequence satisfying $0<a_{k}^{p-1}E{\left|X_{k}\right|}^{p}\leq C<\infty$ for k∈N.*

*(i)*  *If 0<p<1 and $\sum_{k=1}^{\infty }a_{k}$ converges, then*
(2) $$\frac{\sum_{k=1}^{n}a_{k}X_{k}}{\sum_{k=n}^{\infty }a_{k}}\to_{n}0,\;a.s.$$
*(ii)*  *If 1<p≤2 and $E(X_{k})=0,$ $\sup_{n}{\left(\sum_{k=1}^{n}a_{k}\right)}^{-1}\sum_{k=1}^{n}a_{k}E\left|X_{k}\right|<\infty$ and $\sum_{k=1}^{\infty }a_{k}$ diverges, then*
(3) $$\frac{\sum_{k=1}^{n}a_{k}X_{k}}{\sum_{k=1}^{n}a_{k}}\to_{n}0,\;a.s.$$
*(iii)*  *If p>2 and $E(X_{k})=0,$ $\sup_{n}{\left(\sum_{k=1}^{n}a_{k}\right)}^{-1}\sum_{k=1}^{n}a_{k}E\left|X_{k}\right|<\infty$ and $\sum_{k=1}^{\infty }a_{k}$ diverges and $0<a_{k}E{\left|X_{k}\right|}^{2}\leq C<\infty$ for k∈N, then (3) holds.*

**Remark 1.**  *(a) It follows from the proof that in case (i), ψ–mixing is not required.*

*(b) In fact, $\sup_{n}{\left(\sum_{k=1}^{n}a_{k}\right)}^{-1}\sum_{k=1}^{n}a_{k}E\left|X_{k}\right|<\infty$ if $\sup_{k}E\left|X_{k}\right|<\infty$. The latter is also used in the case of pairwise independent random variables (see Theorem 2 in [5]) and can be omitted if $\psi_{m}=0$ for some m≥1.*

*(c) For p=1, Theorem 1 does not hold. Suppose that $\left\{X_{k}\right\}$ is a stochastic sequence $P\left(X_{k}=1\right)=1-P\left(X_{k}=0\right)=1/2$ constructed in the proof of Theorem 1 in [8]. Thus, if $\left\{g_{n}\right\}$ is a non-increasing sequence, $g_{n}\to 0$, then $\psi_{n}≍g_{n}$. Now, let $\left\{\xi_{k}\right\}$ be a sequence of independent random variables with $P\left(\xi_{k}=k\right)=\frac{1}{2k},P\left(\xi_{k}=-k\right)=\frac{1}{2k},P\left(\xi_{k}=0\right)=1-\frac{1}{k}$ and independent of $X_{k}$. Set $Z_{k}=\xi_{k}\cos\left(X_{k}\pi \right)$, k≥1 so that $E\left(e^{itZ_{k}}\right)=E\left(e^{it\xi_{k}}\right)$. Thus, $EZ_{k}=0,E\left|Z_{k}\right|=1$. By Proposition 3.16 on p. 82 in Vol. I, [4] and by Theorem 6.2 on p. 193 in Vol. I, [4] we have that $\left\{Z_{k}\right\}$ is ψ-mixing (non-stationary) with rate $≍g_{n}$. Let $a_{k}\equiv 1.$ Now, (3) fails by the second Borel–Cantelli lemma (see [9] and p. 210 in [10]) since $\sum_{k\geq 1}P(|Z_{k}\left|\geq k\right)=\sum_{k\geq 1}\frac{1}{k}=\infty$.*

The following variant of the converse statement does not require strong mixing.

**Proposition 1.**  *Let $\left\{a_{k}\right\}$ be a sequence of positive reals. Suppose $\left\{X_{k}\right\}$ is an arbitrary dependent random sequence such that $\sup_{k}E\left|X_{k}\right|=C<\infty$ and ${lim inf}_{n}E\left|\sum_{k=1}^{n}a_{k}X_{k}\right|=c>0$. If (3) holds, then $\sum_{k\geq 1}a_{k}$ diverges.*

Set $q=|\frac{p}{p-1}|$. Theorem 1 applied with $a_{k}^{-1}=\left(E\right|X_{k}{{|}^{p})}^{\frac{1}{p-1}}$ and, in case (iii) with $a_{k}^{-1}=E|X_{k}{|}^{2}\vee \left(E\right|X_{k}{{|}^{p})}^{\frac{1}{p-1}}$, yields

**Corollary 1.**  *Suppose $\left\{X_{k}\right\}$ is ψ-mixing.*

*(i)*  *If 0<p<1 and $\sum_{k=1}^{\infty }{∥X_{k}∥}_{p}^{-q}$ converges, then*
(4) $$\frac{\sum_{k=1}^{n}{∥X_{k}∥}_{p}^{-q}X_{k}}{\sum_{n=k}^{\infty }{∥X_{k}∥}_{p}^{-q}}\to_{n}0,\;a.s.$$
*(ii)*  *If 1<p≤2, $\sup_{n}(\sum_{k=1}^{n}∥X_{k}{∥}_{p}^{-q}{)}^{-1}\sum_{k=1}^{n}∥X_{k}{∥}_{p}^{-q}E\left|X_{k}\right|<\infty ,$ $E(X_{k})=0$ and $\sum_{k=1}^{\infty }{∥X_{k}∥}_{p}^{-q}$ diverges, then*
(5) $$\frac{\sum_{k=1}^{n}{∥X_{k}∥}_{p}^{-q}X_{k}}{\sum_{k=1}^{n}{∥X_{k}∥}_{p}^{-q}}\to_{n}0,\;a.s.$$
*(iii)*  *If p≥2,*
$$\underset{n}{\sup}(\sum_{k=1}^{n}∥X_{k}{∥}_{2}^{2}\vee ∥X_{k}{∥}_{p}^{q}{)}^{-1}\sum_{k=1}^{n}(∥X_{k}{∥}_{2}^{2}\vee ∥X_{k}{∥}_{p}^{q}{)}^{-1}E\left|X_{k}\right|<\infty ,$$

*$E(X_{k})=0$ and $\sum_{k=1}^{\infty }(∥X_{k}{∥}_{2}^{2}\vee ∥X_{k}{{∥}_{p}^{q})}^{-1}$ diverges, then*

(6) $$\frac{\sum_{k=1}^{n}(∥X_{k}{∥}_{2}^{2}\vee ∥X_{k}{{∥}_{p}^{q})}^{-1}X_{k}}{\sum_{k=1}^{n}(∥X_{k}{∥}_{2}^{2}\vee ∥X_{k}{{∥}_{p}^{q})}^{-1}}\to_{n}0,\;a.s.$$

We apply our result to the ψ-mixing Cramér model. Namely, we have sequence $\left\{\eta_{k}\right\}$ with $P\left(\eta_{k}=1\right)=1-P\left(\eta_{k}=0\right)=1/\ln k,$ k≥3, which is ψ-mixing. Let $\zeta_{k}=\eta_{k}\ln k$. It is easy to see that $\sigma_{k}^{2}=\mathrm{Var}\left(\zeta_{k}\right)∼\ln k,$ $m_{k}=E\left(\zeta_{k}\right)=1$ and $\sum_{k=3}^{n}\frac{1}{\sigma_{k}^{2}}∼\frac{n}{\ln n}$. Thus, for $\left\{\zeta_{k}\right\}$ in the case p=2, we obtain by (5) that SLLN for Cramér model independence can be replaced by ψ-mixing (see e.g., Lemma A.1 in [11]).

**Lemma 1.** 
$$\lim_{n}\frac{\ln n}{n}\sum_{k=3}^{n}\eta_{k}=1,\;a.s.$$

The paper is organized as follows. In the next section, there are some auxiliary results. These results are required in the proof of Theorem 1 in the last section.

## 2. Auxiliary Results

The following result for p>1 is in [12] and for p=1 see [9] (see also Theorem 2.20 on p. 40 in [13]).

**Theorem 2.**  *Suppose $\left\{X_{k}\right\}$ is ψ-mixing and $E(X_{k})=0,$ k∈Z. Let $\{b_{n}\},$ $b_{0}>0$ be an increasing to infinity sequence of real numbers such that for some p≥1,*

*(i)*  *
$\;\sum_{k=1}^{\infty }b_{k}^{-2p}E(|X_{k}{|}^{2p})<\infty ;$
*
*(ii)*  *$\;\sum_{k=1}^{\infty }b_{k}^{-2}{\left(b_{k}^{2}-b_{k-1}^{2}\right)}^{1-p}{\left(E\left(X_{k}^{2}\right)\right)}^{p}<\infty$;*
*(iii)*  *
$\sup_{n}b_{n}^{-1}\sum_{k=1}^{n}E\left|X_{k}\right|<\infty .$
*

*Then, $b_{n}^{-1}S_{n}\to 0,$ n→∞, almost surely (a.s.).*

The next result improves Lemma 2.1 slightly in [14].

**Proposition 2.**  *Suppose $\left\{X_{k}\right\}$ is ψ-mixing and $E(X_{k})=0,$ k∈Z. Let $b_{n}\in R,$ $b_{0}>0,$ $b_{n}↑\infty ,$ and $\sup_{n}b_{n}^{-1}\sum_{k=1}^{n}E\left|X_{k}\right|<\infty ,$ p∈(1,2], $\sum_{k=1}^{\infty }\frac{E|X_{k}{|}^{p}}{b_{k}^{p}}<\infty ,$ then $b_{n}^{-1}S_{n}\to 0,$ n→∞, a.s.*

**Proof of Proposition 2.**  Set ${\bar{X}}_{k}=X_{k}I(|X_{k}|\leq b_{k})-E(X_{k}I(|X_{k}|\leq b_{k})).$ In view of Theorem 2, we can assume 1<p<2.

$$\sum_{k\geq 1}P(X_{k}\neq X_{k}I(|X_{k}|\leq b_{k}))\leq C\sum_{k\geq 1}\frac{E|X_{k}{|}^{p}}{b_{k}^{p}}<\infty .$$

Further,

$$\begin{array}{c} b_{n}^{-1}|\sum_{k=1}^{n}E(X_{k}I(|X_{k}|\leq b_{k}))|\leq b_{n}^{-1}\sum_{k=1}^{n}E(|X_{k}\left|I(\right|X_{k}|>b_{k}))\\ \;\;\;\leq b_{n}^{-1}\sum_{k=1}^{n}b_{k}^{-p+1}E(|X_{k}{|}^{p}I(|X_{k}|>b_{k}))\leq b_{n}^{-1}\sum_{k=1}^{n}b_{k}^{-p+1}E{\left|X_{k}\right|}^{p}\to 0. \end{array}$$

So that

$$\sum_{k\geq 1}\frac{E{\bar{X}}_{k}^{2}}{b_{k}^{2}}\leq \sum_{k\geq 1}\frac{E(X_{k}^{2}I(|X_{k}|\leq b_{k}))}{b_{k}^{2}}\leq \sum_{k\geq 1}\frac{E|X_{k}{|}^{p}}{b_{k}^{p}}<\infty .$$

Now, apply Theorem 2 to $\left\{{\bar{X}}_{k}\right\}$ with p=1. □

By Lemma 3.6″ on p. 284 in [15] and Kronecker’s lemma (see e.g., [16], p. 236) we have the following SLLN for arbitrary dependent random variables (see also [17]).

**Lemma 2.**  *Suppose $\left\{X_{k}\right\}$ is a sequence of random variables, p∈(0,1], $b_{n}↑\infty$ and $\sum_{k=1}^{\infty }\frac{E|X_{k}{|}^{p}}{b_{k}^{p}}<\infty$. Then, $b_{n}^{-1}S_{n}\to 0,$ n→∞, a.s.*

## 3. Proofs

**Proof of Theorem 1.**  The key role in the proof of Theorem 1 is played by the Dini theorem (see e.g., [18], Theorem 4 on p. 127) and the Abel–Dini theorem (see e.g., [18], Theorem 1 on p. 125) (for a generalization, see Lemma 11 in [7]).

For p∈(0,1) by the Dini theorem,

$$\sum_{n=1}^{\infty }\frac{a_{n}^{p}E{\left|X_{n}\right|}^{p}}{{\left(\sum_{k=n}^{\infty }a_{k}\right)}^{p}}\leq C\sum_{n=1}^{\infty }\frac{a_{n}}{{\left(\sum_{k=n}^{\infty }a_{k}\right)}^{p}}<\infty$$

since $\sum_{k=1}^{\infty }a_{k}<\infty$. Thus, (2) holds by Lemma 2.

Now, assume p∈(1,2]. By the Abel–Dini theorem,

$$\sum_{n=1}^{\infty }\frac{a_{n}^{p}E{\left|X_{n}\right|}^{p}}{{\left(\sum_{k=1}^{n}a_{k}\right)}^{p}}\leq C\sum_{n=1}^{\infty }\frac{a_{n}}{{\left(\sum_{k=1}^{n}a_{k}\right)}^{p}}<\infty$$

since $\sum_{k=1}^{\infty }a_{k}=\infty$. Set $b_{n}=\sum_{k=1}^{n}a_{k}$. Since $\sup_{n}b_{n}^{-1}\sum_{k=1}^{n}a_{k}E\left|X_{k}\right|<\infty ,$

$$\frac{\sum_{k=1}^{n}a_{k}X_{k}}{\sum_{k=1}^{n}a_{k}}\to_{n}0,\;a.s.$$

holds by Proposition 2.

In the case that p>2, set $b_{0}=0.5b_{1}$. By Theorem 2 and the previous point, it is enough to prove that

$$S=\sum_{n=1}^{\infty }\frac{{\left(b_{n}^{2}-b_{n-1}^{2}\right)}^{1-\frac{p}{2}}}{b_{n}^{2}}\frac{a_{n}^{p}{\left(E\left(X_{n}^{2}\right)\right)}^{\frac{p}{2}}}{b_{n}^{p}}<\infty .$$

Let $\delta =\frac{2\epsilon }{p},$ where ϵ∈(0,1) is fixed. In view of $b_{n}\to \infty$, we have that there exist C>0 such that $a_{n}E\left(X_{n}^{2}\right)\leq Cb_{n}^{3-\delta },$ for each *n*. Therefore,

$$a_{n}^{p-1}E{\left(X_{n}^{2}\right)}^{\frac{p}{2}}\leq Cb_{n}^{\frac{3p}{2}-\epsilon }a_{n}^{\frac{p}{2}-1}.$$

But ${\left(b_{n}^{2}-b_{n-1}^{2}\right)}^{1-\frac{p}{2}}={\left(a_{n}b_{n}\left(2-\frac{a_{n}}{b_{n}}\right)\right)}^{1-\frac{p}{2}}$ so that by the Abel–Dini theorem,

$$S\leq C\sum_{n=1}^{\infty }\frac{a_{n}{\left(a_{n}b_{n}\right)}^{1-\frac{p}{2}}}{b_{n}^{p+2}}b_{n}^{\frac{3p}{2}-\epsilon }a_{n}^{\frac{p}{2}-1}=C\sum_{n=1}^{\infty }\frac{a_{n}}{b_{n}^{1+\epsilon }}<\infty .$$

Since $\sup_{n}b_{n}^{-1}\sum_{k=1}^{n}a_{k}E\left|X_{k}\right|<\infty ,$ Theorem 1 is proved. □

**Proof of Proposition 1.**  Suppose $\sum_{k\geq 1}a_{k}<\infty$ and (3) holds. Choose $N_{1}$ such that $\sum_{k\geq n}a_{k}<\frac{c}{3C}$ for $n\geq N_{1}$. Next, choose $N_{2}\geq N_{1}$ such that $E|\sum_{k=1}^{n}a_{k}X_{k}|>c/2$ for $n\geq N_{2}$. Now, for a fixed $N\geq N_{2}$, we have

$$\sum_{k=N+1}^{n}a_{k}X_{k}\to_{n}-\sum_{k=1}^{N}a_{k}X_{k},\;a.s.$$

On the other hand, by Fatou’s lemma (cf. Theorem 5.1 on p. 218, [19]),

$$c/2<E|\sum_{k=1}^{N}a_{k}X_{k}|\leq \underset{n}{lim inf}E\left|\sum_{k=N+1}^{n}a_{k}X_{k}\right|<c/3.$$

Thus, c<0. Contradiction. □

## Data Availability

Data is contained within the article.
